# CRP, IL-1α, IL-1β, and IL-6 levels and the risk of breast cancer: a two-sample Mendelian randomization study

**DOI:** 10.1038/s41598-024-52080-w

**Published:** 2024-01-23

**Authors:** Yongjia Cui, Shasha Cui, Wenping Lu, Ya’nan Wang, Zhili Zhuo, Ruipeng Wang, Dongni Zhang, Xiaoqing Wu, Lei Chang, Xi Zuo, Weixuan Zhang, Heting Mei, Mengfan Zhang

**Affiliations:** Guang An’men Hospital, China Academy of Chinese Medical Sciences, Beijing, 100053 China

**Keywords:** Breast cancer, Cancer prevention

## Abstract

Epidemiological studies have reported a positive association between chronic inflammation and cancer risk. However, the causal association between chronic inflammation and breast cancer (BC) risk remains unclear. Here, we performed a Mendelian randomization study to investigate the etiological role of chronic inflammation in BC risk. We acquired data regarding C-reactive protein (CRP), interleukin (IL)-1a, IL-1b, and IL-6 expression and BC related to single nucleotide polymorphisms (SNPs) from two larger consortia (the genome-wide association studies and the Breast Cancer Association Consortium). Next, we conducted the two-sample Mendelian randomization study to investigate the relationship of the abovementioned inflammatory factors with the incidence of BC. We found that genetically predicted CRP, IL-6, and IL-1a levels did not increase BC incidence (odds ratio (OR)_CRP_ 1.06, 95% confidence interval (CI) 0.98–1.12, *P* = 0.2059, OR_IL-6_ 1.05, 95% CI 0.95–1.16, *P* = 0.3297 and OR_IL-1a_ 1.01, 95% CI 0.99–1.03, *P* = 0.2167). However, in subgroup analysis, genetically predicted IL-1b levels increased ER + BC incidence (OR 1.15, 95% CI 1.03–1.27, *P* = 0.0088). Our study suggested that genetically predicted IL-1b levels were found to increase ER + BC susceptibility. However, due to the support of only one SNP, heterogeneity and pleiotropy tests cannot be performed, which deserves further research.

## Introduction

The number of newly diagnosed breast cancer (BC) cases among women is currently exceeding those of lung cancer which affects women's health^1^. Chronic inflammation has been considered to be associated with tumorigenesis of various cancers such as colorectal cancer^2^, ovarian cancer^3^, and lung cancer^4^, which may be related to the inflammation promoting the formation of an inflammatory microenvironment. In addition, concept of chronic inflammation, prolonged reactive oxygen species production, and activation of stress-linked pathways is considered central to the progression of many inflammatory diseases^5^. Chronic inflammation generates an excess of reactive oxygen and nitrogen species triggering DNA damage and Malignancy^6^. However, the association of chronic inflammation with BC susceptibility remains unknown. C-reactive protein (CRP), a serum marker for chronic inflammation, is produced in the liver under the stimulation of interleukin (IL)-1 and IL-6^7,8^. Studies on the pathological characteristics of many bacterial diseases have revealed that CRP, IL-1, and IL-6 expression in plasma are correlated with each other^9^. CRP, IL-1, and IL-6 have been used as inflammatory markers to investigate the relationship of chronic inflammation with BC susceptibility. The association of CRP with BC susceptibility remains controversial. The researches suggest that increased CRP levels in serum are related to the occurrence of BC^10,11^; in contrast, several clinical studies have shown no relationship between CRP and BC susceptibility^12,13^. Observational studies are inherently prone to confounding and reverse causality, while randomized controlled trials are difficult to carry out. Therefore, it is necessary to further explore the causal relationship between chronic inflammation and the incidence of BC.

IL-1 and IL-6, as upstream stimulators of CRP, have critical roles in BC genesis. IL-1 family members, such as IL-1α and IL-1β, are important factors that mediate chronic inflammation. IL-6 is highly expressed in human BC tissues and cells, and it activates glycoprotein (gp) 130-regulated pathways (MAPK and JAK/STAT pathways) to participate in cellular proliferation, survival, and differentiation. Many in vitro assays have revealed that IL-1 and IL-6 play a role in promoting the growth of BC cells^14–16^. HER2-overexpressing BC cells show enhanced IL-1α production, which acts as a proinflammatory signal to activate additional signaling sequences (such as IL-6) and trigger the STAT3 and NF-κB pathways for generating and maintaining chronic inflammation and cancer stem cells (CSCs), thus promoting tumor occurrence and development^17^. However, there is a lack of clinical studies supporting the association of IL-1/IL-6 with BC susceptibility. Consequently, for providing evidence on the effect of chronic inflammation on BC etiology while minimizing the influence of confounding factors, our present study aimed to explore the causal relationship between CRP, IL-1, IL-6, and BC risk from a genetic perspective.

Recently, Mendelian randomization (MR) method has been proven an effective approach to perform causal association analysis. The concept of MR was first proposed by Katan in 1986; this concept relies on the use of genetic variants that have a strong relationship with exposure and are termed as instrumental variables (IVs) to predict how exposure affects the outcome. After the gametes are formed, they follow the MR inheritance law that parental alleles are randomly allocated to their offspring; thus, the association between genes and outcome remains unchanged even in the presence of confounders such as behavioral factors, puerperal environment, reverse causality, and socioeconomic status^18,19^. A previously published genome-wide association study (GWAS) provides an opportunity for a two-sample MR (TSMR) study that can improve statistical power and precision. TSMR uses two-sample summary data, which represent exposure-associated and outcome-related genetic variations.

Based on the role of chronic inflammation in the etiology of BC disease, we performed a TSMR analysis to assess the causal relationship between CRP, IL-1α, IL-1β, and IL-6 levels and BC risk. A preprint has previously been published^20^.

## Methods

### Exposure data sources

Summary data for CRP, IL-6, IL-1a, and IL-1b-related single nucleotide polymorphisms (SNPs) were down from the GWAS summary data (https://gwas.mrcieu.ac.uk/). The GAWS IDs are ieu-b-35, prot-a-1539, GCST006621, and prot-a-1495. The CRP-related SNPs were found from a meta-analysis of GWASs including 204,402 European individuals from 88 studies^21^. The IL-6-and IL-1b-related SNPs were studied from the INTERVAL study, including 3,301 normal subjects, nested based on 50,000 blood donors’ genetic biological resources at 25 centers in England^22^. The IL-1a-associated SNPs were derived from the genetic study of up to 700 maternal and infant cytokines/chemokines^23^.

### Summary data on BC

According to the expression of estrogen receptor (ER), BC is divided into ER + BC and ER − BC, which exhibit different biological behaviors. Thus, they will be discussed in subgroups. Summary data for BC-associated SNPs, including 122,977 cases (69,501 ER + cases and 21,468 ER − cases) and 105,974 controls, were acquired from the Breast Cancer Association Consortium (BCAC)^24^. The summary data was downloaded from the GWAS database (https://gwas.mrcieu.ac.uk/), and the corresponding IDs are ieu-a-1126, ieu-a-1127, and ieu-a-1128.

### Statistical analyses

**Figure 1:**
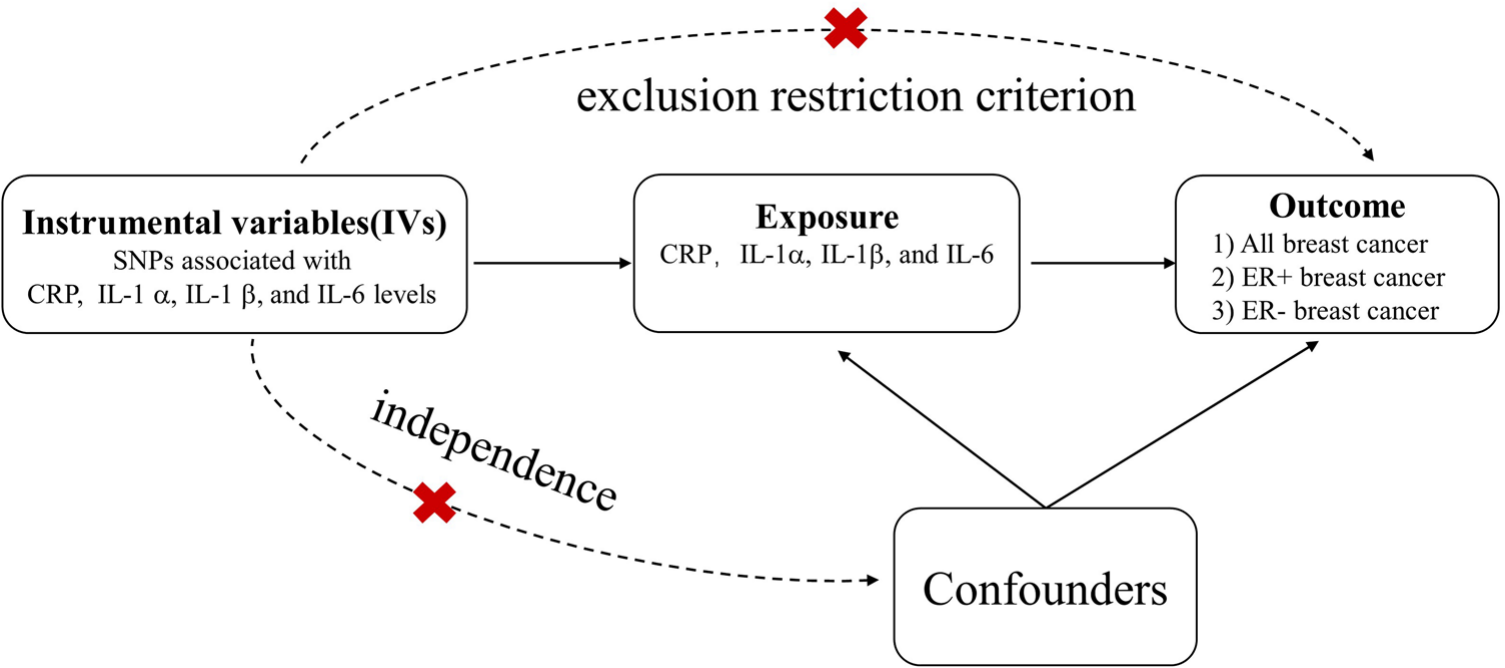
Basic assumptions in designing MR study. *SNP* single nucleotide polymorphism, *CRP* C-reactive protein.

The present study is a TSMR study. As shown in Fig. 1, the research of this study is based on three hypotheses: (1) IVs and exposure factors are strongly correlated (association); (2) IVs and confounders are not correlated (independence); and (3) IVs and the outcome are not directly related, and the effect on the outcome can only be demonstrated by exposure (exclusion restriction criterion). In a TSMR study, exposure-related IVs and outcome-related IVs are obtained from two independent samples (e.g., a GWAS of exposure-related SNPs and a GWAS of outcome-related SNPs) from the same ethnic group. Compared to a single-sample Mendelian randomization study, TSMR involves a larger sample size and thus can obtain greater power. Currently, TSMR is widely used because of the availability of public data from a large number of GWAS collaborative groups worldwide.

To select the IVs that are strongly correlated with exposures (first MR assumption), this study implemented quality control for selecting potentially helpful SNPs. First, a genome-wide significance analysis was conducted to identify SNPs related to exposures (IL-1α, IL-1β, IL-6, and CRP) (*P* < 5e−08). Second, the exposure-related helpful SNPs should not be within linkage disequilibrium (LD), because SNPs strongly related to LD possibly induce bias in outcomes. The present work implemented a clumping procedure (r^2^ < 0.001, threshold of distance = 10,000 kb). Third, the low instrumental bias was assessed by F statistic, where F statistic of < 10 confirms that the selected genetic variants do not meet the strong correlation between IVs and exposure^25^. F statistic was calculated using the formula: Beta2/SE2, where beta and se represent genetic association with the exposure and standard deviation, respectively. In addition, when choosing the IVs for exposures, the following conditions also need to be considered: First, we eliminated SNPs whose minor allele frequency (MAF) was lower than 0.01 in the process of extracting outcome information from IVs. Second, we eliminated recurrent SNPs from those chosen helpful SNPs during harmonization for ensuring that the effect of SNPs on exposure and the effect of identical SNPs on outcome were corresponding to the identical allele.

For CRP-associated SNPs, inverse-variance weighted (IVW), weighted median, and MR-Egger approaches were used for inferring causality, the most important of which was the IVW method. IVW is a method for meta-aggregating the effects of multiple loci in the process of MR analysis^26^. The application of IVW is based on the concept that all SNPs are valid IVs and are completely independent of each other. Based on IVW, we additionally modified the MR-Egger approach. Compared to the IVW method, the core of this method is to consider the existence of the intercept term in the weighted linear regression. Simultaneously, the intercept term is used to measure the pleiotropy among the IVs, and the slope estimates the causal effect in an unbiased manner^27^. The weighted median method is the median values obtained by sorting all individual SNP effect values by weight. Weighted median can be a robust estimate when at least 50% of the genetic variation meets the MR core assumptions^28^. For IL-6-, IL-1α-, and IL-1β-associated SNPs, we used the Wald ratio for determining estimates for one SNP^29^.

### Sensitivity analysis

To satisfy independence of the MR study (second MR assumption), after a comprehensive lookup of the PhenoScanner (http://www.phenoscanner.medschl.cam.ac.uk/), the significant associations of the selected SNPs with BC risk factors (*P* < 1e−08) were excluded, including BRCA1/2 gene mutation, childbearing, breastfeeding, mammographic density, height, obesity, alcohol intake, physical inactivity, age of menarche, and menopause^30^.

Experimental conditions, analytical platforms, and different study subjects may contribute to heterogeneity, leading to biased causal effect estimates. To meet the exclusion restriction criterion of the MR study (third MR assumption), based on MR-Egger and IVW analyses, our study used the Cochran *Q* statistic to conduct a heterogeneity test for detecting heterogeneity in causal estimate. *P* > 0.05 indicated the absence of heterogeneity within those enrolled IVs, and thus, we applied the fixed-effects MR estimates in such models; else, we used the random-effects MR estimates.

When MR-Egger, IVW, Wald ratio, and weighted median approaches were used to investigate causality, there may be other unknown confounders that were not conducive to biased estimates of genetic diversity and causal effects. We determined horizontal pleiotropy based on the intercept of MR-Egger and its *P* values. If the MR-Egger regression intercept was close to 0 (< 0.1) and *P* > 0.05, we considered that there was no evidence of horizontal pleiotropy in the test. Mendelian randomization pleiotropy residual sum and outlier (MR-PRESSO) method was used for better validating potential outliers and horizontal pleiotropy^31^. We set 5000 distributions as the parameter during MR-PRESSO analyses.

After the assessment of heterogeneity and horizontal pleiotropy, we performed a sensitivity analysis of the eligible SNPs by using the leave-one-out method. This method aims to re-estimate the causal effect by sequentially removing one SNP at each time in order to determine the SNP that greatly affects causal effect estimates. The results are considered to be reliable if the overall error bars do not change significantly after excluding each SNP.

### Statistical power

According to the online calculation method (https://shiny.cnsgenomics.com/mRnd/) of the MR statistical power reported by Brion et al.^32^, α (type I error) was 0.05 and K (case composition ratio) was 54%. To calculate R^2^ for the SNP instrument, we used the following formula: 2 × EAF × (1-EAF) × beta^2^^33^, where EAF and beta represent effect allele frequency and predicted genetic impact on exposure, respectively. R^2^xz (proportion of variance associated with CRP explained by SNPs) was 3.98%, while R^2^xz (proportion of variance associated with IL-6 explained by SNPs) was 1.06%. Based on the meta-analysis of clinical observational trials, the RR value of CRP for BC in the European population was OR = 1.12^34^, and the risk ratio of IL-6 for BC was OR = 1.13^35^. We used the number of BC summary data (n = 228,951, 122,977 cases, and 105,974 controls) as the sample size. Our MR study had a high power (≥ 80%), and the results are shown in Supplementary Table S3. Statistical power for IL-1α and IL-1β was not calculated because of the lack of clinical studies on the association between IL-1α and IL-1β levels and BC risk.

The present study used the GWAS data to verify whether there was a causal relationship between CPR, IL-1α, IL-1β, and IL-6 levels and BC risk. All data were available online, and the data were analyzed with TwoSampleMR package version 0.5.6 and R version 4.1.0. *P* < 0.05 was considered to be statistically significant. The multiple testing refers to multiple statistical tests on the same data set, which increases the probability of Type I error. Therefore, we adjust the test level using the Bonferroni method with an adjusted test level of α = 0.05/number of statistical tests. There are four exposure factors in our paper (CRP, IL-6, IL-1α, IL-1β), so the link between exposure and outcome phenotype is considered to be statistically significant when the Bonferroni-corrected *P* value is < 0.0125.

## Results

### MR estimates for CRP, IL-1α, IL-1β, and IL-6 levels with BC risk

#### MR estimates for CRP and BC susceptibility

There was almost no evidence that suggested the association of genetically predicted CRP level with all BC (Table 1) (OR 1.05, 95% CI 0.98–1.12, *P* = 0.2059), ER + BC risk (OR 1.07, 95% CI 0.98–1.17, *P* = 0.1340), and ER-BC (OR 1.04, 95% CI 0.94–1.15, *P* = 0.4396). Twelve SNPs were excluded by a comprehensive lookup of the PhenoScanner (Table 2). Two SNPs (rs4656849 and rs7121935) were excluded after removing LD. Three palindromic SNPs (rs2293476, rs10240168, and rs6485751) were excluded. F-statistics for CRP-associated SNPs ranged from 53 to 2408, indicating a strong correlation between IVs and exposure. CRP-associated SNPs could explain 3.98% of the total genetic variation. Supplementary Table S1 shows detailed information on CRP-associated SNPs. Supplementary Table S2 presents the summarized results of CRP and BC. Supplementary Figs.S1–S6 exhibit forest and scatter plots for the relationship of CRP with BC susceptibility.

**Table 1:** The MR estimate results of CRP, IL-1, IL-1, and IL-6 association with breast cancer risk.

Exposure	nsnp	All breast cancer		ER + breast cancer		ER − breast cancer	
		OR (95% CI)	*P* value	OR (95% CI)	*P* value	OR (95% CI)	*P* value
CRP							
MR-Egger	36	0.99 (0.89,1.11)	0.8989	1.03 (0.90,1.18)	0.6868	1.00 (0.86.1.17)	0.9721
Weighted median	36	1.02 (0.96,1.09)	0.4946	1.07 (0.99,1.16)	0.0870	1.05 (0.93.1.19)	0.3956
Inverse variance weighted	36	1.05 (0.98,1.12)	0.2059	1.07 (0.98,1.17)	0.1340	1.04 (0.94.1.15)	0.4396
IL-1α							
Wald ratio	1	1.01 (0.99,1.03)	0.2167	1.01 (0.99,1.03)	0.2509	1.00 (0.97,1.03)	0.8569
IL-1β							
Wald ratio	1	1.07 (0.98,1.17)	0.1140	1.15 (1.03,1.27)	0.0088	1.00 (0.86,1.18)	0.9510
IL-6							
Wald ratio	1	1.05 (0.95,1.16)	0.3297	1.08 (0.95,1.22)	0.2312	0.97 (0.81,1.16)	0.7155

*MR* Mendelian randomization, *CRP* C-reactive protein, *IL-1α* interleukin-1α, *IL-1β* interleukin-1β, *IL-6* interleukin-6, *nsnp* number of single nucleotide polymorphism, *OR* odds ratio, *CI* confidence interval.

**Table 2:** Twelve SNPs excluded by a comprehensive lookup of the PhenoScanner.

SNPs	Trait in PhenoScanner
rs1260326	Type II diabetes^46^, Height^47^,Alcohol consumption^48^,Age at menopause^49^
rs12995480	Overweight^50^, Age at menarche^51^
rs1490384	Type II diabetes adjusted for BMI^52^, Height^53^, Menarche age at onset^54^, Age at menarche^51^
rs3134899	Height^55^
rs1558902	Menarche age at onset^56^, Overweight^50^,Type II diabetes^57^
rs12960928	Type II diabetes^52^, Height^47^, Overweight^50^
rs4420638	Type II diabetes^58^
rs12202641	Height^47^
rs10832027	Age at menarche^51^
rs7310409	Height^53^, Type II diabetes^46^
rs1800961	Type II diabetes^46^
rs6001193	Age at menopause^49^

#### MR estimates for IL-1α with BC risk

Weak evidence was noted for the association of genetically predicted IL-1α level with all breast cancer (Table 1) (OR 1.01, 95% CI 0.99–1.03, *P* = 0.2167). In subgroup analysis, little evidence supported that IL-1α was related to ER + BC susceptibility (OR 1.01, 95% CI 0.99–1.03, *P* = 0.2509) and ER-BC susceptibility (OR 1.00, 95% CI 0.97–1.03, *P* = 0.8569). F-statistics for the IL-1α-associated SNP was 33.67, indicating that the SNP was unlikely to be affected by the weak instrument bias. IL-1α-associated SNPs could explain 2.38% of the total genetic variation. Genome-wide significant SNP loci for IL-1α along with F-statistics and R^2^ values are shown in Supplementary Table S1. The summary data for IL-1α and BC are shown in Supplementary Table S2.

#### MR estimates for IL-1β with BC risk

The results indicate a significant P-value in the investigation of the relationship between IL-1β and ER + BC risk (OR 1.15, 95% CI 1.03–1.27, *P* = 0.0088) (Table 1), however, it is supported by only one SNP, and there are not enough SNPs to conduct assessment of heterogeneity and horizontal pleiotropy. In such a case, we should be more cautious in interpreting the relationship between IL-1β and ER + BC risk. Inverse results were found with all BC (OR 1.07, 95% CI 0.98–1.17, *P* = 0.1140) and ER-BC (OR 1.00, 95% CI 0.86–1.18, *P* = 0.9510). One palindromic SNP (rs13402561) was excluded. IL-1β associated with SNP whose F-statistics was 34.20, was strongly correlated with exposure. The IL-1β-associated SNP could explain 1.02% of the total genetic variation. Genome-wide significant SNP loci for IL-1β along with F-statistics and R^2^ are shown in Supplementary Table S1. Summary data for IL-1β and BC are presented in Supplementary Table S2.

#### MR estimates for IL-6 with BC risk

MR estimates showed no significant relationship between genetically predicted IL-6 level and all BC (Table 1) (OR 1.05, 95% CI 0.95–1.16, *P* = 0.3297). Subgroup analysis demonstrated less evidence of a causal relationship between IL-6 and ER + BC risk (OR 1.08, 95% CI 0.95–1.22, *P* = 0.2312) or ER-BC risk (OR 0.97, 95% CI 0.81–1.16, *P* = 0.7155). F-statistics for the IL-6-associated SNP was 31, indicating that the SNP was unlikely to be affected by weak instrument bias. The IL-6-associated SNPs could explain 1.06% of the total genetic variation. Genome-wide significant SNP loci for IL-6 along with F-statistics and R^2^ values are shown in Supplementary Table S1. Summary data for IL-6 and BC are presented in Supplementary Table S2.

### Sensitivity analyses

Heterogeneity test showed some significant differences for causal estimation between CRP level and all BC risk (IVW, Q (*df*) 86.04^35^, *P* = 3.47E−06; MR-Egger, Q (*df*) 82.29^34^, *P* = 6.97E−06), ER + BC (IVW, Q (*df*) 95.04^35^, *P* = 1.89E−07; MR-Egger, Q(*df*) 93.60^34^, *P* = 1.79E−07), and ER-BC (IVW, Q (*df*) 50.06^35^, *P* = 0.0475; MR-Egger, Q (*df*) 49.54^34^, *P* = 0.0414).Thus, random-effects MR estimates were applied to these models. Heterogeneity test could not be performed for IL-1α, IL-1β, and IL-6 because of only one SNP.

Regarding horizontal pleiotropy in MR estimates for CRP and BC, the results demonstrated no evidence of horizontal pleiotropy effects based on the evaluation by the MR-Egger intercept and its *P* values (All BC, intercept = 0.0036, *P* = 0.2220; ER + BC, intercept = 0.0026, *P* = 0.4745; ER-BC, intercept = 0.0024, *P* = 0.5527). The MR-PRESSO method did not find any potential pleiotropy in MR estimation of a causal relationship between CRP level and BC risk, but it found some outliers in all BC or ER + BC. For all BC, following the exclusion of SNPs (rs13233571) in the restrictive MR analysis, a significant causal relationship between CRP and all BC risk was not observed (OR 1.06, 95% CI 0.99–1.13, *P* = 0.1020, Table 3). The leave-one-out, scatter plot and forest plot were shown in Supplementary Figs.S7–S9. For ER + BC, following the exclusion of SNPs (rs1051338, rs2064009, and rs9271608) in the restrictive MR analysis, a similar result was observed for ER + BC (OR 1.06, 95% CI 0.99–1.15, *P* = 0.1119, Table 3). The leave-one-out, scatter plot and forest plot were shown in Supplementary Figs. S10–S12.Outlier SNPs were not found after the application of MR-PRESSO method in ER-BC. Because exposure has only one SNP as the IV, which included IL-1α, IL-1β, and IL-6, the MR-Egger intercept and MR-PRESSO methods could not be used for evaluating horizontal pleiotropy. However, we searched the PhenoScanner website and found no correlation between these IVs and other confounding factors.

**Table 3:** Results of the MR-PRESSO method applied after excluding CRP genetic variants associated with BC risk (*P* < 0.05).

	All BC^a^				ER + BC^b^			
	Beta	SE	*P* value	OR (95% CI)	Beta	SE	*P* value	OR (95% CI)
IVW (initial)	0.0457	0.0362	0.2059	1.05 (0.98,1.12)	0.0683	0.0456	0.1340	1.07 (0.98,1.17)
IVW (after outlier removal)	0.0558	0.0341	0.1020	1.06 (0.99,1.13)	0.0608	0.0382	0.1119	1.06 (0.99,1.15)

*IVW* inverse-variance weighted, *SE* standard error, *OR* odds ratio, *CI* confidence interval.

ER-BC absence of Outlier SNPs.

^a^Outlier SNPs: rs13233571.

^b^Outlier SNPs: rs1051338, rs2064009, rs9271608.

**Figure 2:**
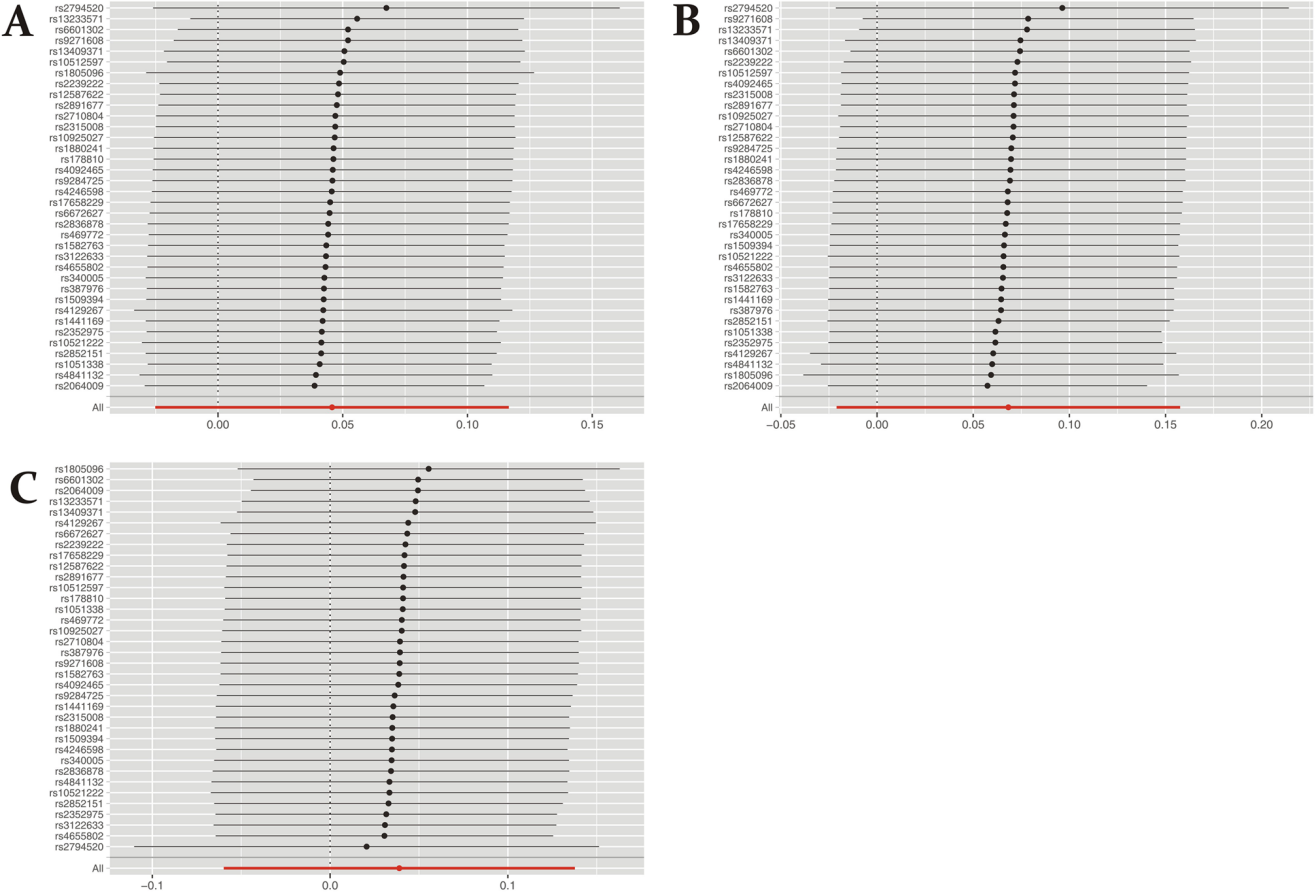
MR sensitivity analyses for the association between CRP and BC. (**A**) All BC. (**B**) ER + BC. (**C**) ER − BC. Regardless of which SNP was removed, all points were to the right side of 0, indicating that the MR results were very robust.

The results of the Leave-one-out sensitivity test showed that regardless of which CRP-associated SNP was removed, no significant change was observed in the results, indicating that the MR results were very robust (Fig. 2). Unfortunately, the Leave-one-out sensitivity test could not be used to verify the robustness of IL-1α, IL-1β, and IL-6 because of only one SNP.

## Discussion

Our TSMR analysis of more than 122,977 patients with BC together with 105,974 normal controls suggested that CRP, IL-1α, and IL-6 did not play an etiological role in BC susceptibility. In subgroup analyses, some evidence was observed suggesting that genetically predicted IL-1β expression may increase susceptibility to ER + BC, but this finding is based on just one SNP and lacks heterogeneity and horizontal pleiotropy analysis. Sensitivity analyses also showed that IVs affected outcomes only through exposure, rather than confounding and other pathways; thus, implying no pleiotropy.

The research on the relationship between CRP and the incidence of cancer, including colon cancer, BC, lung cancer, etc., has been widely studied^2–4,8–11^. In the exploration of BC incidence and CRP, prospective observational clinical studies have shown contradictory results^8–11^. The impossibility of random controlled trials has hindered the research on the relationship between CRP and BC risk. In our study using Mendelian Randomization to analyze the relationship between CRP and BC risk, we found no causal relationship, which is consistent with previous research results^36,37^. We calculated Cochran's Q to quantify the heterogeneity of the causal effects, and a *P* value ≤ 0.05 indicated the presence of heterogeneity, thus necessitating the use of random-effects IVW MR analysis^38^. We also used MR-Egger regression based on the intercept term to assess the potential presence, where a *P* value ≥ 0.05 indicated no gene pleiotropy ^39^. Additionally, the slope of the MR-Egger regression provided effective MR estimates in the presence of horizontal pleiotropy. We also used the weighted-median method to calculate OR estimates, which can provide valid MR estimates even when up to 50% of the included instruments are invalid^28^. The IVW, MR-Egger, and the weighted-median method failed to find a causal relationship between CRP and BC incidence. Using the Leave-one-out method to observe the impact of remaining SNPs after excluding individual SNPs one by one, no significant SNPs were found, indicating robust results^40^. We also used MR-PRESSO to assess the presence of pleiotropy^31^. In this case, after excluding outlier SNPs, the results did not change completely. In summary, our results demonstrate there is not a causal relationship between CRP and BC incidence. We speculate that CRP is not a direct cause of BC but rather a reactant to other risk factors that can lead to chronic inflammation, which requires further investigation.

The “upstream” proinflammatory factors IL-1/IL-6 play an essential role in initiating chronic inflammation which further triggers the production of inflammatory factors, including CRP. The expression level of IL-6 is significantly higher by 2.3-fold in BC patients compared to healthy individuals^41^. Serum IL-6 level is closely related to the degree of tumor infiltration, lymph node metastasis, distant metastasis, and TNM stage^42^. IL-6 is suggested to accelerate BC progression in most cellular assays; however, our study did not find a relationship between genetically predicted elevation of IL-6 and increased BC susceptibility; thus, showing consistency with previous studies^43^. Similar results were obtained from a meta-analysis on the association of IL-6 gene polymorphisms with BC susceptibility^44^. Previous studies have also confirmed that IL-6 gene polymorphisms are associated with BC susceptibility^45^, possibly because of different races^46^.

According to our results, genetically predicted IL-1β levels increased ER + BC risk. However, due to the support of only one SNP, heterogeneity and gene pleiotropy tests cannot be performed, and we interpret the results with greater caution. IL-1α or IL-1β levels are elevated in the serum of BC patients and are associated with an aggressive disease phenotype such as advanced or metastatic^47^. To the best of our knowledge, the present MR work is the first to investigate the relationship of IL-1β content with BC susceptibility. The IL-1β-related SNP in our paper is located on the SARM1 gene. Further regulation comes from sterile-α and armadillo motif containing protein (SARM) that negatively regulates the assembly of the NLRP3 (NOD-, LRR- and pyrin domain-containing 3) inflammasome, suppressing the maturation of IL-1β^48^. It suggests that the SARM gene influences the level of IL-1β. Both ER + BC cells and ER-BC cells show different IL-1β signaling pathways, which may be due to different IL-1 receptors on the cell surface^49^. IL-1β directly affects the transcriptional activation of ER-alpha^50^. IL-1 dysregulation contributes to the occurrence, development, and migration of cancers, and therefore, IL-1 blockers have become increasingly popular in clinical trials of patients with cancers. Anakinra (Kineret), a recombinant, nonglycosylated IL-1Ra that negatively regulates IL-1α and IL-1β, was used to treat patients with metastatic BC (NCT01802970); however, the results have not yet been published. IL-1α is less stimulatory than IL-1β in stimulating liver CRP production; however, it appears to trigger chronic inflammation by stimulating the NF-κB pathway^51^. In our present study, no causal relationship was observed between IL-1α and BC susceptibility. This is different from the results of previous studies^46^, which might be due to different study populations.

Our present study has some limitations. First, we selected European ancestry as our objects for reducing population stratification bias; consequently, the results cannot be applied to populations with different genetic backgrounds. Second, we were unable to stratify patients according to menopausal status or severity of CRP because of the lack of individual data. Third, we used only genetic tools to assess the causal relationship between inflammatory biomarkers and BC risk. Finally, we only investigated the causal relationship between CRP, IL-1, IL-6 expression levels and breast cancer risk, without analyzing other biomarkers of chronic inflammation and transcription factors, which are also crucial for breast cancer occurrence. More animal or human trials are needed to explore the relationship between chronic inflammation and breast cancer occurrence. Therefore, our results need to be treated with caution, as individuals can adapt to genetic changes through compensatory mechanisms, and the etiological role of other inflammatory factors in BC needs to be further investigated.

## Conclusion

Our present study indicated that genetically predicted IL-1β levels increase the susceptibility of ER + BC, whereas the levels of CRP, IL-1α, and IL-6 were not related to BC susceptibility. To the best of our knowledge, the present MR study is the first to investigate the relationship of IL-1β with BC susceptibility, and the results suggest the different etiological effects of IL-1β on ER + BC as compared to that on ER-BC, which deserves further study.

### Supplementary Information

Supplementary Information.

## Data Availability

All data generated or analysed during this study are included in this published article and its supplementary information files. The GWAS summary statistics for CRP, IL-1β, IL-6, and breast cancer (including ER + breast cancer and ER− breast cancer) is available in the the OpenGWAS database(https://gwas.mrcieu.ac.uk/). The GWAS summary statistics for IL-1α is available in the GWAS Catalog (https://www.ebi.ac.uk/gwas/).
