# Supplementary material for: CRP, IL-1α, IL-1β, and IL-6 levels and the risk of breast cancer: a two-sample Mendelian randomization study
Source: Sci Rep. 2024 Jan 23;14:1982. doi: 10.1038/s41598-024-52080-w (PMC10805756; doi:10.1038/s41598-024-52080-w)
Supplement: Supplementary file 1 — Supplementary Information. [file 41598_2024_52080_MOESM1_ESM.docx]

***Supporting Information***

**Supplemental Table S1** Genome-wide significant SNPs loci association with CRP, IL6, IL1α, and IL1β for Mendelian randomization on breast cancer

**Supplemental Table S2** The Effect Allele Alignment Result for exposures and outcomes for MR analyses. (Exposures including CRP, IL6, IL1α, IL1β, outcomes including all breast cancer, ER+ breast cancer, ER- breast cancer)

**Supplementary Table S3** Power assessment to validate previously observed risk from potential risk parameters using Mendelian randomization analysis

**Supplementary Figure S1** MR scatter plot for CRP on Overall Breast Cancer

**Supplementary Figure S2** MR forest plot for CRP on Overall Breast Cancer

**Supplementary Figure S3** MR scatter plot for CRP on ER+ Breast Cancer

**Supplementary Figure S4** MR forest plot for CRP on ER+ Breast Cancer

**Supplementary Figure S5** MR scatter plot for CRP on ER- Breast Cancer

**Supplementary Figure S6** MR forest plot for CRP on ER- Breast Cancer

**Supplementary Figure S7** MR leave−one−out sensitivity analysis from MR-PRESSO applied after excluding genetic variants for CRP on Overall Breast Cancer

**Supplementary Figure S8** MR scatter plot from MR-PRESSO applied after excluding genetic variants for CRP on Overall Breast Cancer

**Supplementary Figure S9** MR forest plot from MR-PRESSO applied after excluding genetic variants for CRP on Overall Breast Cancer

**Supplementary Figure S10** MR leave−one−out sensitivity analysis from MR-PRESSO applied after excluding genetic variants for CRP on ER+ Breast Cancer

**Supplementary Figure S11** MR scatter plot from MR-PRESSO applied after excluding genetic variants for CRP on ER+ Breast Cancer

**Supplementary Figure S12** MR forest plot from MR-PRESSO applied after excluding genetic variants for CRP on ER+ Breast Cancer

**Supplemental Table S1** Genome-wide significant SNPs loci association with CRP, IL6, IL1α, and IL1β for Mendelian randomization on breast cancer

| SNP | Chr | Pos | Effect allele | Other allele | Beta | SE | EAF | P-value | N^a^ | R^2^ | F-statistic |
| --- | --- | --- | --- | --- | --- | --- | --- | --- | --- | --- | --- |
| CRP | | | | | | | | | | | |
| rs1805096 | 1 | 66102257 | A | G | -0.104381 | 0.003614 | 0.38625 | 2.17E-183 | 204402 | 0.005165744 | 834.1931321 |
| rs6672627 | 1 | 154346967 | A | C | -0.037135 | 0.005083 | 0.146148 | 2.89E-13 | 204402 | 0.000344169 | 53.37361727 |
| rs4129267 | 1 | 154426264 | T | C | -0.087519 | 0.003612 | 0.385524 | 1.20E-129 | 204402 | 0.003629034 | 587.0961199 |
| rs3122633 | 1 | 159377458 | C | T | 0.027479 | 0.00389 | 0.301705 | 1.68E-12 | 204402 | 0.000318166 | 49.90024128 |
| rs2293476* | 1 | 40036847 | C | G | 0.030262 | 0.004225 | 0.225941 | 8.27E-13 | 204402 | 0.000320328 | 51.30288962 |
| rs4655802 | 1 | 65888231 | A | G | -0.025012 | 0.00416 | 0.614793 | 1.88E-09 | 204402 | 0.000296312 | 36.15015625 |
| rs469772 | 1 | 91530305 | T | C | -0.031327 | 0.004542 | 0.193541 | 5.54E-12 | 204402 | 0.000306354 | 47.57111759 |
| rs2794520 | 1 | 159678816 | T | C | -0.182186 | 0.003712 | 0.333842 | 1.00E-200 | 204402 | 0.014763122 | 2408.873902 |
| rs4656849*** | 1 | 159723521 | G | A | 0.057656 | 0.003723 | 0.621343 | 4.91E-54 | 204402 | 0.001564215 | 239.8296898 |
| rs10925027 | 1 | 247612562 | C | T | -0.036035 | 0.00382 | 0.599644 | 4.25E-21 | 204402 | 0.000623475 | 88.98613148 |
| rs9284725 | 2 | 102744854 | A | C | -0.02731 | 0.00419 | 0.761212 | 7.34E-11 | 204402 | 0.000271139 | 42.4830173 |
| rs1260326** | 2 | 27730940 | C | T | -0.073462 | 0.003604 | 0.607156 | 2.72E-92 | 204402 | 0.002574399 | 415.4855565 |
| rs1509394 | 2 | 28647084 | T | C | 0.025685 | 0.004147 | 0.542959 | 6.05E-10 | 204402 | 0.000327425 | 38.36110153 |
| rs13409371 | 2 | 113838145 | A | G | 0.048232 | 0.003847 | 0.429854 | 5.07E-36 | 204402 | 0.00114027 | 157.1904543 |
| rs12995480** | 2 | 629881 | C | T | 0.031261 | 0.004855 | 0.830925 | 1.24E-10 | 204402 | 0.000274585 | 41.45980588 |
| rs4246598 | 2 | 88438050 | A | C | 0.022063 | 0.003547 | 0.46258 | 5.11E-10 | 204402 | 0.000242025 | 38.69071478 |
| rs1441169 | 2 | 214033530 | G | A | -0.024926 | 0.003725 | 0.532568 | 2.27E-11 | 204402 | 0.000309335 | 44.77675607 |
| rs2352975 | 3 | 49891885 | C | T | 0.024897 | 0.004026 | 0.303573 | 6.43E-10 | 204402 | 0.000262097 | 38.24251956 |
| rs17658229 | 5 | 172191052 | C | T | 0.055568 | 0.009522 | 0.045881 | 5.50E-09 | 204402 | 0.000270343 | 34.05596397 |
| rs9271608 | 6 | 32591588 | G | A | 0.042021 | 0.004954 | 0.215335 | 2.33E-17 | 204402 | 0.000596708 | 71.94833734 |
| rs12202641 | 6 | 116314634 | T | C | -0.022804 | 0.003617 | 0.39442 | 3.00E-10 | 204402 | 0.000248418 | 39.74889382 |
| rs1490384** | 6 | 126851160 | T | C | -0.024816 | 0.003545 | 0.512283 | 2.65E-12 | 204402 | 0.000307731 | 49.0039493 |
| rs3134899** | 6 | 31473286 | T | C | 0.023329 | 0.004274 | 0.767055 | 4.93E-08 | 204402 | 0.000194492 | 29.79361563 |
| rs13233571 | 7 | 72971231 | T | C | -0.056895 | 0.005476 | 0.119609 | 2.95E-25 | 204402 | 0.000681738 | 107.9496714 |
| rs1880241 | 7 | 22759469 | G | A | -0.027537 | 0.003687 | 0.48295 | 8.41E-14 | 204402 | 0.000378702 | 55.78108711 |
| rs2710804 | 7 | 36084529 | C | T | 0.021262 | 0.003737 | 0.367409 | 1.30E-08 | 204402 | 0.000210141 | 32.37144098 |
| rs10240168* | 7 | 22819334 | G | C | -0.028684 | 0.004343 | 0.221389 | 4.11E-11 | 204402 | 0.000283652 | 43.62141698 |
| rs2891677 | 8 | 126344208 | T | C | 0.019859 | 0.003511 | 0.538958 | 1.59E-08 | 204402 | 0.000195993 | 31.992862 |
| rs6601302 | 8 | 9239458 | G | T | -0.030518 | 0.004478 | 0.736931 | 9.80E-12 | 204402 | 0.000361109 | 46.44553383 |
| rs2064009 | 8 | 117007850 | T | C | 0.027111 | 0.003549 | 0.581445 | 2.28E-14 | 204402 | 0.000357752 | 58.35513462 |
| rs4841132 | 8 | 9183596 | G | A | 0.065095 | 0.006243 | 0.909023 | 2.00E-25 | 204402 | 0.000700861 | 108.719787 |
| rs644234 | 9 | 136142217 | G | T | 0.022597 | 0.003708 | 0.369203 | 1.13E-09 | 204402 | 0.000237841 | 37.13830857 |
| rs1051338 | 10 | 91007360 | G | T | 0.023881 | 0.003992 | 0.308119 | 2.27E-09 | 204402 | 0.000243156 | 35.78688947 |
| rs1582763 | 11 | 60021948 | A | G | -0.022107 | 0.0037 | 0.37181 | 2.37E-09 | 204402 | 0.000228298 | 35.69901015 |
| rs7121935*** | 11 | 72496148 | A | G | -0.021853 | 0.00374 | 0.376337 | 5.28E-09 | 204402 | 0.000224171 | 34.14121143 |
| rs6485751* | 11 | 47336442 | C | G | -0.03089 | 0.004359 | 0.218471 | 1.43E-12 | 204402 | 0.00032584 | 50.2183083 |
| rs10832027** | 11 | 13357183 | A | G | 0.025944 | 0.003745 | 0.668809 | 4.43E-12 | 204402 | 0.000298184 | 47.99215231 |
| rs11108056 | 12 | 95855385 | G | C | -0.027907 | 0.003708 | 0.42371 | 5.42E-14 | 204402 | 0.000380335 | 56.64307915 |
| rs4767920 | 12 | 121065277 | A | G | -0.038519 | 0.0049 | 0.842127 | 4.00E-15 | 204402 | 0.000394517 | 61.79564186 |
| rs10778215 | 12 | 103537266 | A | T | -0.033242 | 0.003583 | 0.488517 | 1.86E-20 | 204402 | 0.000552224 | 86.07572195 |
| rs7310409** | 12 | 121424861 | G | A | 0.137075 | 0.003706 | 0.607808 | 1.00E-200 | 204402 | 0.008958012 | 1368.061683 |
| rs2239222 | 14 | 73011885 | G | A | 0.035484 | 0.003901 | 0.363541 | 9.87E-20 | 204402 | 0.000582665 | 82.73956638 |
| rs12587622 | 14 | 73365174 | A | G | -0.020798 | 0.003609 | 0.500861 | 8.52E-09 | 204402 | 0.000216278 | 33.21003885 |
| rs340005 | 15 | 60878030 | A | G | 0.030007 | 0.003736 | 0.620695 | 1.01E-15 | 204402 | 0.000423977 | 64.51065054 |
| rs10521222 | 16 | 51158710 | T | C | -0.104411 | 0.010714 | 0.04533 | 2.06E-22 | 204402 | 0.000943543 | 94.97060977 |
| rs1558902** | 16 | 53803574 | A | T | 0.033926 | 0.003701 | 0.405653 | 5.20E-20 | 204402 | 0.000554996 | 84.02860338 |
| rs178810 | 17 | 16097430 | T | C | 0.02001 | 0.003606 | 0.562924 | 2.95E-08 | 204402 | 0.000197029 | 30.79234277 |
| rs10512597 | 17 | 72699833 | C | T | 0.036931 | 0.00489 | 0.817288 | 4.44E-14 | 204402 | 0.000407337 | 57.03801678 |
| rs12960928** | 18 | 57897803 | C | T | 0.024 | 0.003993 | 0.267003 | 1.91E-09 | 204402 | 0.000225461 | 36.12633152 |
| rs2852151 | 18 | 12841176 | A | G | 0.024735 | 0.003655 | 0.404527 | 1.36E-11 | 204402 | 0.000294757 | 45.79826933 |
| rs4092465 | 18 | 55080437 | G | A | 0.027483 | 0.004364 | 0.650698 | 3.11E-10 | 204402 | 0.000343351 | 39.66055542 |
| rs4420638** | 19 | 45422946 | G | A | -0.229459 | 0.006122 | 0.17962 | 1.00E-200 | 204402 | 0.015517078 | 1404.829255 |
| rs387976 | 19 | 45379060 | C | A | 0.025507 | 0.003946 | 0.331946 | 1.05E-10 | 204402 | 0.000288554 | 41.78347947 |
| rs1800961** | 20 | 43042364 | T | C | -0.1115 | 0.011267 | 0.033816 | 4.63E-23 | 204402 | 0.000812385 | 97.93392175 |
| rs2315008 | 20 | 62343956 | G | T | 0.023467 | 0.003777 | 0.687731 | 5.36E-10 | 204402 | 0.000236533 | 38.60301068 |
| rs2836878 | 21 | 40465534 | A | G | -0.042902 | 0.004079 | 0.27418 | 7.71E-26 | 204402 | 0.000732571 | 110.6235692 |
| rs6001193** | 22 | 39074737 | G | A | -0.027809 | 0.003706 | 0.35065 | 6.53E-14 | 204402 | 0.000352171 | 56.30667914 |
| IL6 |  |  |  |  |  |  |  |  |  |  |  |
| rs11872808 | 18 | 4331629 | T | C | 0.2297 | 0.0411 | 0.11345 | 2.18776E-08 | 3301 | 0.010613527 | 31.23477247 |
| IL1α |  |  |  |  |  |  |  |  |  |  |  |
| rs1562064 | 4 | 146313100 | G | A | -0.74 | 0.12755102 | 0.32 | 2.00E-08 | 228951 | 0.23831552 | 33.65856256 |
| IL1β |  |  |  |  |  |  |  |  |  |  |  |
| rs13402561* | 2 | 3639921 | G | C | -0.3247 | 0.0326 | 0.81414 | 2.19E-23 | 3301 | 0.031906532 | 99.20404419 |
| rs967645 | 17 | 26713970 | T | C | -0.1427 | 0.0244 | 0.50945 | 5.13E-09 | 3301 | 0.010178008 | 34.20332236 |

SNP single nucleotide polymorphism, Chr chromosome, Pos Position, SE standard error, EAF effect allele frequency.

a N refers to the sample size of the initial GWAS from which the genetic variants were selected

* removing the SNPs for being palindromic

** Confounding related SNPs by a comprehensive lookup of the PhenoScanner

*** removing the SNPs for linkage disequilibrium

**Supplemental Table S2** The Effect Allele Alignment Result for exposures and outcomes for MR analyses. (Exposures including CRP, IL6, IL1α, IL1β, outcomes including all breast cancer, ER+ breast cancer, ER- breast cancer)

|  |  |  | | **All breast cancer** | | | | | **ER+ breast cancer** | | | | **ER-breast cancer** | | | |
| --- | --- | --- | --- | --- | --- | --- | --- | --- | --- | --- | --- | --- | --- | --- | --- | --- |
| **SNP** | **Effect allele** | **Other allele** | | **Beta** | | **EAF** | **SE** | **P-value** | **Beta** | **EAF** | **SE** | **P-value** | **Beta** | **EAF** | **SE** | **P-value** |
| **CRP** | | | | | | | | | | | | | | | | |
| rs10512597 | C | T | -0.013 | | 0.8139 | | 0.0079 | 0.1022 | -0.0085 | 0.8139 | 0.0095 | 0.3676 | -0.0053 | 0.8149 | 0.0145 | 0.7149 |
| rs1051338 | G | T | 0.0201 | | 0.2986 | | 0.007 | 0.00399797 | 0.0269 | 0.2991 | 0.0083 | 0.001242 | -0.0064 | 0.2988 | 0.0127 | 0.617999 |
| rs10521222 | T | C | -0.0261 | | 0.0434 | | 0.0168 | 0.1196 | -0.0193 | 0.0434 | 0.0199 | 0.3307 | -0.0305 | 0.0439 | 0.03 | 0.3094 |
| rs10925027 | C | T | 6.00E-04 | | 0.5987 | | 0.0064 | 0.9209 | 0.0031 | 0.5985 | 0.0076 | 0.684699 | 0.0014 | 0.5985 | 0.0116 | 0.9047 |
| rs12587622 | A | G | 0.0073 | | 0.4994 | | 0.0062 | 0.2371 | 0.0065 | 0.4999 | 0.0074 | 0.3763 | 0.0082 | 0.4984 | 0.0113 | 0.4637 |
| rs13233571 | T | C | 0.0356 | | 0.1117 | | 0.0108 | 0.000982291 | 0.0327 | 0.1109 | 0.0129 | 0.0110999 | 0.0312 | 0.1127 | 0.0193 | 0.1064 |
| rs13409371 | A | G | -0.0057 | | 0.3912 | | 0.0065 | 0.3744 | -0.0065 | 0.3914 | 0.0077 | 0.3961 | -0.0122 | 0.3915 | 0.0118 | 0.3005 |
| rs1441169 | G | A | -0.012 | | 0.5278 | | 0.0062 | 0.0543701 | -0.0124 | 0.5284 | 0.0074 | 0.0946608 | -0.0106 | 0.5276 | 0.0113 | 0.3479 |
| rs1509394 | T | C | 0.0115 | | 0.5512 | | 0.0065 | 0.0783105 | 0.0087 | 0.5526 | 0.0078 | 0.2623 | 0.0135 | 0.5528 | 0.0119 | 0.2562 |
| rs1582763 | A | G | -0.0093 | | 0.371 | | 0.0065 | 0.1485 | -0.0139 | 0.3702 | 0.0077 | 0.07284 | -0.001 | 0.3703 | 0.0118 | 0.9312 |
| rs17658229 | C | T | 0.01 | | 0.0403 | | 0.017 | 0.5587 | 0.017 | 0.04 | 0.0204 | 0.4025 | -0.0272 | 0.0402 | 0.0316 | 0.3891 |
| rs178810 | T | C | -0.001 | | 0.5647 | | 0.0062 | 0.8733 | 0.0036 | 0.565 | 0.0075 | 0.627401 | -0.0068 | 0.5651 | 0.0114 | 0.5478 |
| rs1805096 | A | G | -0.0028 | | 0.3823 | | 0.0063 | 0.661301 | -0.0127 | 0.3832 | 0.0076 | 0.0920598 | 0.006 | 0.3813 | 0.0115 | 0.6042 |
| rs1880241 | G | A | 3.00E-04 | | 0.4744 | | 0.0062 | 0.9664 | 0.0017 | 0.4729 | 0.0074 | 0.8171 | -0.0113 | 0.4748 | 0.0113 | 0.3206 |
| rs2064009 | T | C | 0.021 | | 0.5874 | | 0.0064 | 0.00109999 | 0.0326 | 0.5878 | 0.0077 | 2.26E-05 | -0.0285 | 0.5866 | 0.0117 | 0.01472 |
| rs2239222 | G | A | -0.0051 | | 0.3585 | | 0.0068 | 0.4588 | -0.0091 | 0.359 | 0.0082 | 0.2695 | -0.0069 | 0.3592 | 0.0124 | 0.5754 |
| rs2315008 | G | T | -0.0035 | | 0.6927 | | 0.0067 | 0.6053 | -0.0088 | 0.694 | 0.008 | 0.2708 | 0.0143 | 0.6935 | 0.0122 | 0.2416 |
| rs2352975 | C | T | 0.0158 | | 0.3101 | | 0.0069 | 0.0215199 | 0.0254 | 0.3091 | 0.0082 | 0.00203601 | 0.0272 | 0.3103 | 0.0126 | 0.03122 |
| rs2710804 | C | T | -0.0039 | | 0.371 | | 0.0065 | 0.5427 | -0.0082 | 0.3707 | 0.0077 | 0.2896 | -5.00E-04 | 0.3718 | 0.0118 | 0.9666 |
| rs2794520 | T | C | -0.0028 | | 0.3374 | | 0.0065 | 0.6625 | -0.0053 | 0.3376 | 0.0078 | 0.4992 | -0.0118 | 0.338 | 0.0119 | 0.3216 |
| rs2836878 | A | G | -0.006 | | 0.2632 | | 0.0078 | 0.4433 | -8.00E-04 | 0.2631 | 0.0093 | 0.93 | -0.0135 | 0.2647 | 0.014 | 0.3369 |
| rs2852151 | A | G | 0.0142 | | 0.4055 | | 0.0063 | 0.0249799 | 0.0168 | 0.4058 | 0.0075 | 0.0251901 | 0.0191 | 0.4051 | 0.0115 | 0.09742 |
| rs2891677 | T | C | -0.0062 | | 0.5445 | | 0.0063 | 0.3237 | -0.0092 | 0.5448 | 0.0075 | 0.2182 | -0.0079 | 0.5444 | 0.0115 | 0.490399 |
| rs3122633 | C | T | 0.0089 | | 0.3032 | | 0.0068 | 0.1892 | 0.0104 | 0.3032 | 0.008 | 0.1977 | 0.0259 | 0.3022 | 0.0123 | 0.03509 |
| rs340005 | A | G | 0.0097 | | 0.6216 | | 0.0066 | 0.1433 | 0.0069 | 0.6211 | 0.0079 | 0.3829 | 0.0128 | 0.6214 | 0.012 | 0.2842 |
| rs387976 | C | A | 0.0125 | | 0.3455 | | 0.0069 | 0.0686199 | 0.0148 | 0.3463 | 0.0082 | 0.0713296 | -2.00E-04 | 0.3488 | 0.0125 | 0.9875 |
| rs4092465 | G | A | 3.00E-04 | | 0.6313 | | 0.007 | 0.9684 | -0.0096 | 0.6312 | 0.0084 | 0.2525 | 0.003 | 0.6324 | 0.0127 | 0.8121 |
| rs4129267 | T | C | -0.0067 | | 0.3923 | | 0.0063 | 0.2889 | -0.0121 | 0.3913 | 0.0076 | 0.1105 | 5.00E-04 | 0.3909 | 0.0116 | 0.9686 |
| rs4246598 | A | C | 0.0015 | | 0.4531 | | 0.0068 | 0.824 | -0.0027 | 0.4533 | 0.0081 | 0.742699 | 0.0166 | 0.4561 | 0.0122 | 0.1748 |
| rs4655802 | A | G | -0.0102 | | 0.5995 | | 0.0068 | 0.1352 | -0.0108 | 0.5999 | 0.0082 | 0.1855 | -0.0298 | 0.5992 | 0.0124 | 0.01607 |
| rs469772 | T | C | -0.0079 | | 0.1861 | | 0.0083 | 0.3419 | -0.0033 | 0.1854 | 0.0099 | 0.740099 | 0.0044 | 0.1869 | 0.015 | 0.7678 |
| rs4841132 | G | A | 0.026 | | 0.9101 | | 0.0112 | 0.0195501 | 0.0338 | 0.9099 | 0.0133 | 0.0112401 | 0.0224 | 0.9103 | 0.0205 | 0.2729 |
| rs6601302 | G | T | 0.0218 | | 0.7538 | | 0.0077 | 0.00483704 | 0.0202 | 0.7541 | 0.0093 | 0.0298298 | 0.0379 | 0.7544 | 0.0142 | 0.007425 |
| rs6672627 | A | C | -0.0058 | | 0.1433 | | 0.0091 | 0.5253 | -0.0041 | 0.1425 | 0.0109 | 0.706801 | 0.0166 | 0.1428 | 0.0164 | 0.3103 |
| rs9271608 | G | A | -0.0185 | | 0.1693 | | 0.0085 | 0.0297502 | -0.03 | 0.1685 | 0.0102 | 0.00310599 | 9.00E-04 | 0.1693 | 0.0162 | 0.954 |
| rs9284725 | A | C | -7.00E-04 | | 0.7579 | | 0.0073 | 0.9275 | 0.0036 | 0.758 | 0.0087 | 0.6807 | -0.0104 | 0.757 | 0.0132 | 0.4315 |
| **IL6** | | | | | | | | | | | | | | | | |
| rs11872808 | T | C | | 0.0116 | | 0.0971 | 0.0119 | 0.33 | 0.017 | 0.0964 | 0.0142 | 0.2307 | -0.0078 | 0.0977 | 0.0214 | 0.715201 |
| **IL1α** | | | | | | | | | | | | | | | | |
| rs1562064 | G | A | | -0.0084 | | 0.3001 | 0.0068 | 0.2127 | -0.0093 | 0.3009 | 0.0081 | 0.2473 | 0.0022 | 0.3042 | 0.0122 | 0.8596 |
| **IL1β** | | | | | | | | | | | | | | | | |
| rs967645 | T | C | | -0.0098 | | 0.5124 | 0.0062 | 0.1167 | -0.0194 | 0.5125 | 0.0074 | 0.00885299 | -0.0007 | 0.5129 | 0.0114 | 0.9503 |

SNP single nucleotide polymorphism, SE standard error, EAF effect allele frequency.

**Supplementary Table S3** Power assessment to validate previously observed risk from potential risk parameters using Mendelian randomization analysis

| Exposures | **Previous observed risk increase** | | **Power assessment** | |
| --- | --- | --- | --- | --- |
|  | OR (95% CI) | Publication | OR (95% CI) | Mendelian randomization power (%) |
| CRP | 1.12(1.02,1.23) | GUO L et al. 2015 | 1.06(0.97,1.15) | 1 |
| IL6 | 1.13(0.94,1.37) | Il'yasova D et al. 2005 | 1.05(0.95,1.16) | 0.85 |

Statistical power for IL1α, IL1β was not calculated due to lack of clinical studies on IL1α, IL1β and breast cancer risk.

**
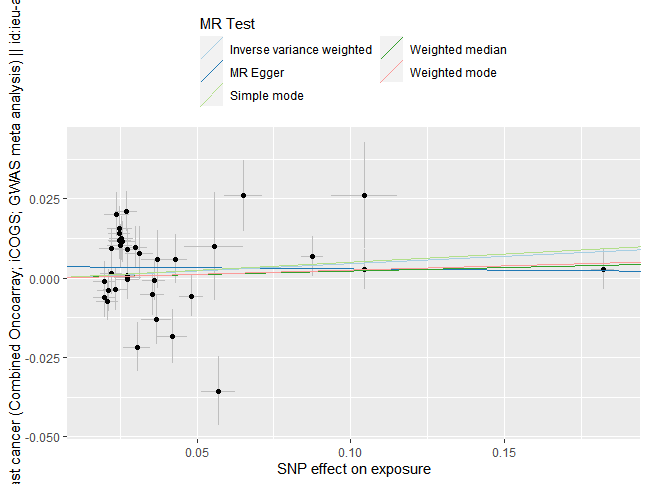
**

**Supplementary Figure S1** MR scatter plot for CRP on Overall Breast Cancer

**
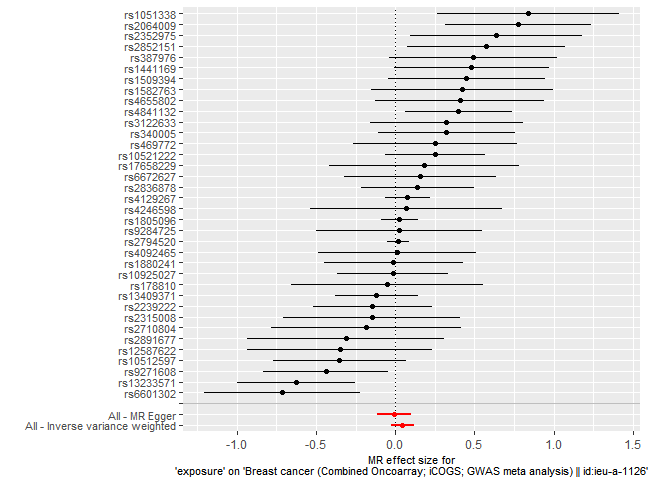
**

**Supplementary Figure S2** MR forest plot for CRP on Overall Breast Cancer

**
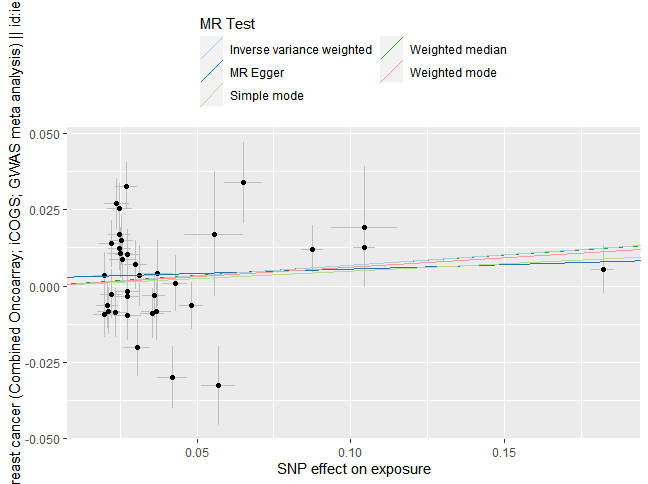
**

**Supplementary Figure S3** MR scatter plot for CRP on ER+ Breast Cancer

**
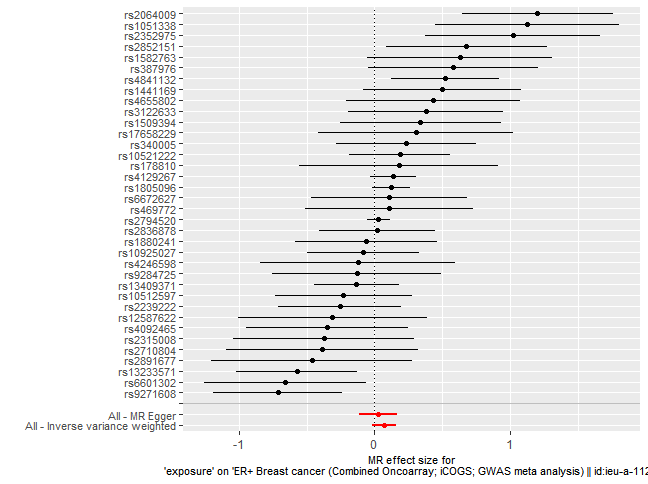
**

**Supplementary Figure S4** MR forest plot for CRP on ER+ Breast Cancer

**
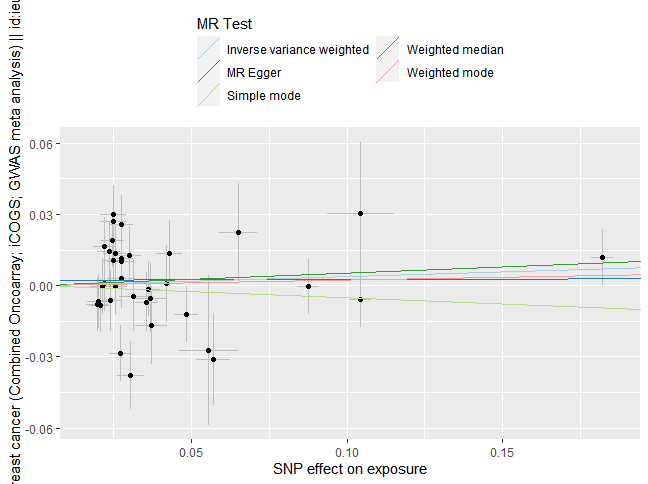
**

**Supplementary Figure S5** MR scatter plot for CRP on ER- Breast Cancer

**
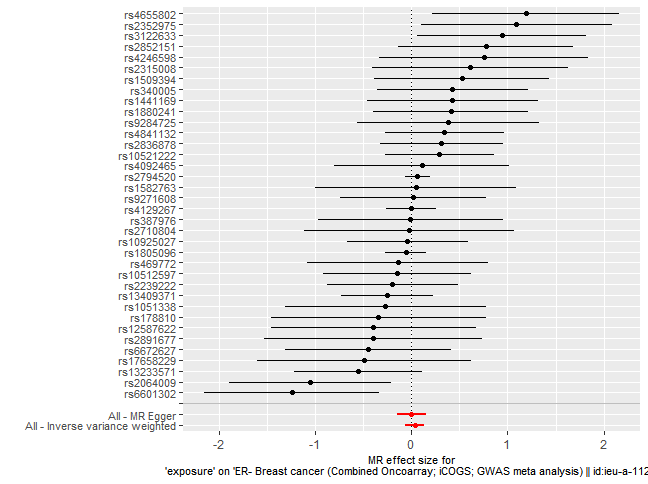
**

**Supplementary Figure S6** MR forest plot for CRP on ER- Breast Cancer

**
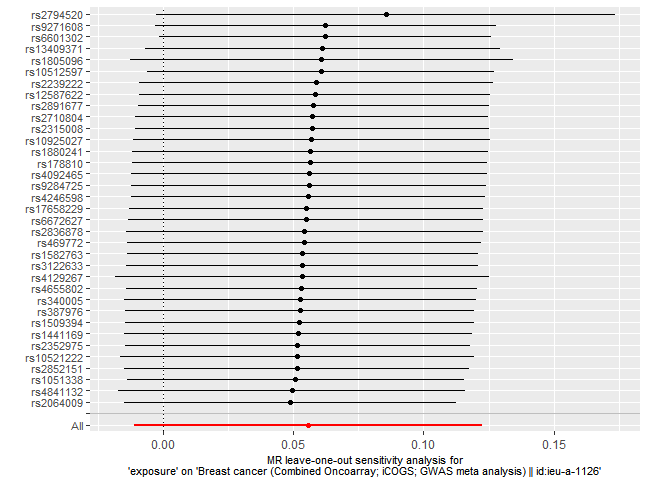
**

**Supplementary Figure S7** MR leave−one−out sensitivity analysis from MR-PRESSO applied after excluding genetic variants for CRP on Overall Breast Cancer

**
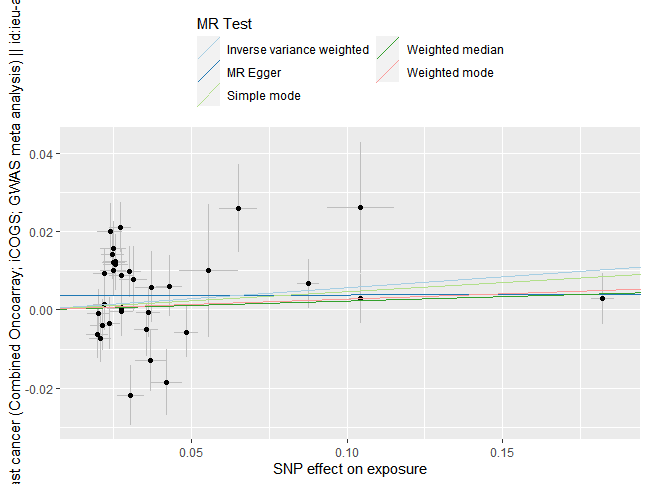
**

**Supplementary Figure S8** MR scatter plot from MR-PRESSO applied after excluding genetic variants for CRP on Overall Breast Cancer

**
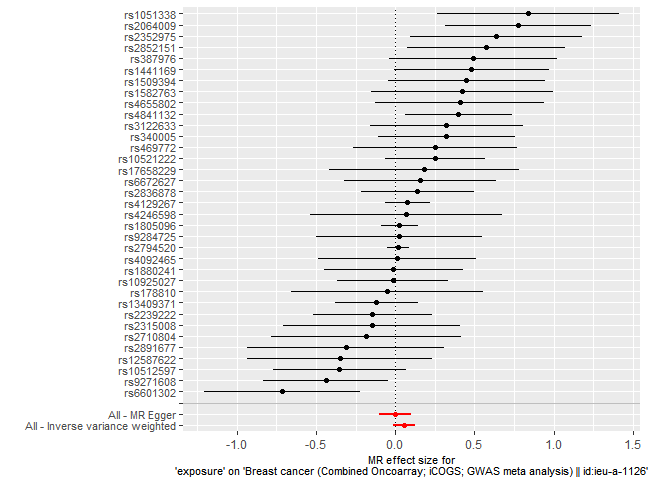
**

**Supplementary Figure S9** MR forest plot from MR-PRESSO applied after excluding genetic variants for CRP on Overall Breast Cancer

**
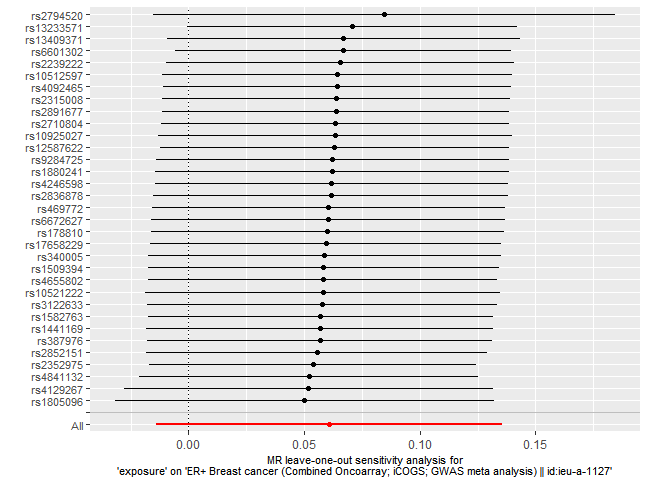
**

**Supplementary Figure S10** MR leave−one−out sensitivity analysis from MR-PRESSO applied after excluding genetic variants for CRP on ER+ Breast Cancer

**
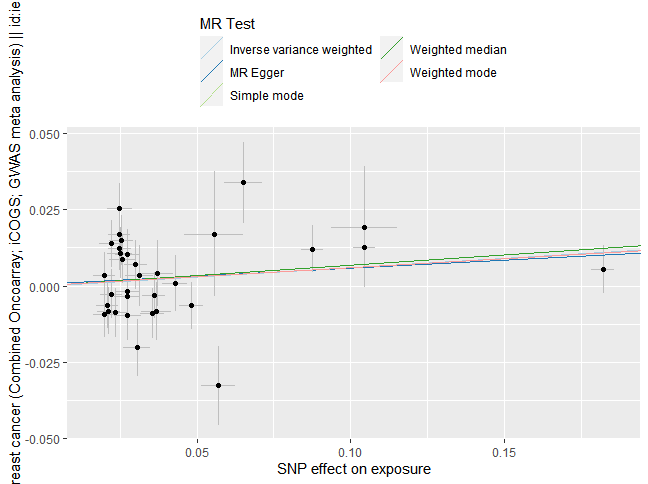
**

**Supplementary Figure S11** MR scatter plot from MR-PRESSO applied after excluding genetic variants for CRP on ER+ Breast Cancer

**
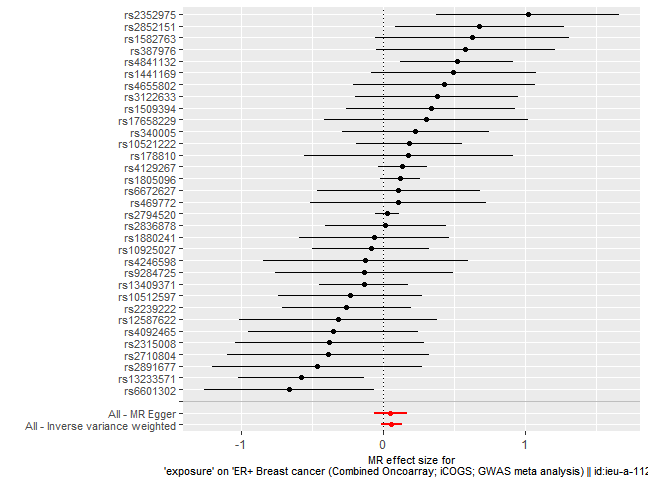
**

**Supplementary Figure S12** MR forest plot from MR-PRESSO applied after excluding genetic variants for CRP on ER+ Breast Cancer
